# BSocial: Deciphering Social Behaviors within Mixed Microbial Populations

**DOI:** 10.3389/fmicb.2017.00919

**Published:** 2017-05-24

**Authors:** Jessica Purswani, Rocío C. Romero-Zaliz, Antonio M. Martín-Platero, Isabel M. Guisado, Jesús González-López, Clementina Pozo

**Affiliations:** ^1^Environmental Microbiology Group, Institute of Water Research, University of GranadaGranada, Spain; ^2^Department of Microbiology, University of GranadaGranada, Spain; ^3^M4Mlab, Department of Computer Science and Artificial Intelligence, University of GranadaGranada, Spain

**Keywords:** biofilms, high throughput, microbial interactions, net-positive species, social behavior, microbial cooperation, bioremediation, microbial fitness

## Abstract

Ecosystem functionality depends on interactions among populations, of the same or different taxa, and these are not just the sum of pairwise interactions. Thus, know-how of the social interactions occurring in mixed-populations are of high interest, however they are commonly unknown due to the limitations posed in tagging each population. The limitations include costs/time in tediously fluorescent tagging, and the number of different fluorescent tags. Tag-free strategies exist, such as high-throughput sequencing, but ultimately both strategies require the use of expensive machinery. Our work appoints social behaviors on individual strains in mixed-populations, offering a web-tool (**BSocial** http://m4m.ugr.es/BSocial.html) for analyzing the community framework. Our quick and cheap approach includes the periodic monitoring of optical density (OD) from a full combinatorial testing of individual strains, where number of generations and growth rate are determined. The BSocial analyses then enable us to determine how the addition/absence of a particular species affects the net productivity of a microbial community and use this to select productive combinations, i.e., designate their social effect on a general community. Positive, neutral, or negative assignations are applied to describe the social behavior within the community by comparing fitness effects of the community against the individual strain. The usefulness of this tool for selection of optimal inoculum in biofilm-based methyl *tert*-butyl ether (MTBE) bioremediation was demonstrated. The studied model uses seven bacterial strains with diverse MTBE degradation/growth capacities. Full combinatorial testing of seven individual strains (triplicate tests of 127 combinations) were implemented, along with MTBE degradation as the desired function. Sole observation of highest species fitness did not render the best functional outcome, and only when strains with positive and neutral social assignations were mixed (*Rhodococcus ruber* EE6, *Agrobacterium* sp. MS2 and *Paenibacillus etheri* SH7), was this obtained. Furthermore, the use of positive and neutral strains in all its combinations had a significant higher degradation mean (x1.75) than exclusive negative strain combinations. Thus, social microbial processes benefit bioremediation more than negative social microbial combinations. The BSocial webtool is a great contributor to the study of social interactions in bioremediation processes, and may be used in other natural or synthetic habitat studies.

## Introduction

Mixed microbial populations can be found in a variety of niches, from aquatic systems as planktonic cells (Kim et al., 2014), to oral cavities in the form of biofilms (Bortolaia and Sbordone, 2002). Biofilms form in the interfaces of different density states, and have been found in “extreme” environments such as glacier-fed streams (Wilhelm et al., 2013) or near hydrothermal vents (Hall-Stoodley et al., 2004). Their resistance to external changes make biofilms the potentially leading bacterial-state for bioremediation (Fortin and Deshusses, 1999; Kharoune et al., 2001; Purswani et al., 2014). One of the most important decisions when selecting a microbial inoculum is whether to use an existing mixed-microbial population (e.g., sludge from a prior existing bioreactor) or select pure microbial cultures. In the latter case, if only one strain is used, this strain must be able to perform efficiently under the conditions provided. The use of more than one strain may offer more resilience to change and higher functional rates through cooperating genomes; however the opposite may also occur through nutrient competition (Saleem et al., 2016) or production of antagonistic compounds (Saleem et al., 2012). The stability-diversity hypothesis states that the more diverse a community is, the more stable and productive it becomes. In natural systems, single microbial species habitats have not been reported, however “extreme” or poisonous environments may contain low species richness. However, natural biofilms are rarely composed of solely two microbial strains, so how can we test individual social behaviors from a highly diverse community?

The net productivity of a biofilm composed of mixed microbial populations is subject to the individual's capacity, the public goods availability (a nutrient produced by a microbe and available to other microbes) and the social interaction with its neighbors. Functional outcome from social interactions among microorganisms can be inferred when two individual microorganisms are studied (whether genetically similar, or from different taxa). According to Hamilton (1964), social effects have direct fitness consequences i.e., affect the rate of cell division.

Modeling and experimental work have being generally done on the competition of up to four genetically different individuals (Katsuyama et al., 2009; Nadell et al., 2009; Lee et al., 2014; Ren et al., 2015). Nevertheless, in the environment, functional outcome of mixed microbial population systems of >2 strains are not held by the sum of the social pairwise interactions as Hamilton describes in his social interaction matrix. In order to find the cost and benefits for the actor and the receiver within a biofilm, competition assays of the individuals are to be done. However, there is no cheap and easy way to do this since it would require tedious fluorescent tagging of each strain (limited to the amount of different fluorescent tags), or high-throughput sequencing as an alternative tag-free strategy, both which require the use of expensive machinery. Another method which has been performed recently includes monitoring individual and four-species consortium from seven different soil isolates to study cooperative forces through the observation of biofilm biomass production, enabling the identification of a consortium obtaining high biofilm biomass production (Ren et al., 2015). An individual species with strong dominance (97% relative abundance) in the culture was described within the latter consortium, and strain qPCR monitoring in each combinatorial of these four strains described the importance of each strain to obtain the highest biofilm biomass production in the four-species consortium. Interestingly, Fetzer et al. (2015) studied the interactions of a 12 species community and their possible individual role in a community. Microbial consortia tested were (benzoate) degrader and non-degrader strains. Only 20% of all possible combinations were tested, and function (growth) was determined as change in biofilm biomass production (ΔOD) using two points in time (0 and 72 h). General positive growth tendencies were observed with increasing species richness, although the rates were different for each environment tested. The impact of individual species on the different communities were also tested, resulting in definition of species that impact positively, negatively or with no effect toward the general community.

In this paper, we present a high throughput analytical method that enables testing of large numbers of strain combinations, and analyses via a webtool for deciphering behavior in microbial social interactions (BSocial), by assessing the net interaction of the focal species with the rest of the microbial community. The use of growth rate and number of generations generated were used as measures of fitness, and MTBE degradation as the functional unit to describe the community performance. The social behavior of each strain tested is assessed in two different environments (methyl *tert*-butyl ether—MTBE, ethyl *tert*-butyl ether—ETBE) both with a same biochemical degradation pathway. Through this approach we have determined the fittest and most functional community through the mixture of socially cooperative microbes.

## Materials and methods

### Chemicals

All chemicals purchased were of reagent grade or of the highest purity available. The fuel ether MTBE (99.9% purity) and ETBE (99% purity) were purchased from Sigma-Aldrich (Milwaukee, WI, USA), and are used in this study to measure community fitness in different xenobiotic compounds.

### Growth medium

The growth media used in the experiments was a mineral salts medium (Purswani et al., 2011) with the following composition: KH_2_PO_4_, 0.225 g/L; K_2_HPO_4_, 0.225 g/L; (NH_4_)_2_SO_4_, 0.225 g/L; MgSO_4_·7H_2_O, 0.050 g/L; CaCO_3_, 0.005 g/L; FeCl_2_·4H_2_O, 0.005 g/L; and 1 ml of trace elements solution. Trace elements solution had the following composition: ZnSO_4_·7H_2_O, 0.1 g/L; MnCl_2_·4H_2_O, 0.03 g/L; H_3_BO_3_, 0.3 g/L; CoCl_2_·6H_2_O, 0.2 g/L; CuCl_2_·2H_2_O, 0.01 g/L; NiCl_2_·6H_2_O, 0.02 g/L; and NaMoO_4_·2H_2_O, 0.03 g/L. Moreover the medium was supplemented with 1 mL vitamin solution whose composition was: biotin 20 μg/L; folic acid 20 μg/l; pyridoxine HCL 100 μg/L; thiamine HCL 50 μg/L; riboflavin 50 μg/L; nicotinic acid 50 μg/L; dipantotenato calcium 50 μg/L; p-aminobenzoic calcium 50 μg/L; lipoicacid 50 μg/L; and cobalamin 50 μg/L.

### Microorganisms used in this study

The strains used were previously isolated based on their growth, degrading and/or tolerance capacities on methyl *tert*-butyl ether (MTBE) under laboratory conditions, and these were: *Rhodococcus ruber* A5, *Nocardia nova* A6, *R. ruber* DD1, *Stenotrophomonas* sp. DD8, *R. ruber* EE6, *Agrobacterium* sp. MS2, and *Paenibacillus etheri* SH7^T^ (Guisado et al., 2013, 2015, 2016; Purswani et al., 2014). The function, observed in our HT-Growth method as growth yield and rate in different xenobiotic compounds, will help determine which bacterial consortium is suitable as an inoculum for a biofilm-based remediating reactor.

### High throughput growth (HT-growth)

The use of sterile closed-lid 96-well polystyrene flat-bottom plates (VACUTEST Kima S.r.l) for growth of biofilms previously used in other systems (Coenye and Nelis, 2010), allows for the maximum number of different combinations from total number of strains. For our study, we used seven strains, making it a total of 127 combinations, which we could fit easily onto two 96-well plates. Strain combinations were prepared with equal optical densities (0.2 OD_595nm_) and equal volumes (30 μl) for each strain, and were all adjusted to contain a final volume of 300 μl. A volume of 300 μl 60% glycerol was mixed with the 300 μl strain combination, and 20 μl aliquots of the latter were placed onto replicate 96-well plates and stored at −20°C. For the growth experiments, 180 μl of mineral medium with vitamins (solely, or amended with final 150 mg/L MTBE or ETBE) was added and mixed by pipetting. Growth at OD λ = 595 nm of all strain combinations were measured (at 1 h intervals) in triplicate for each condition tested, using a spectrophotometer (FluoStar Optima, BMG–Labtech) with heating lids at 30°C. Defining the consortium using direct fitness measured in rate of cell division or No. generations was intended to allow the BSocial user more information of its consortia. Fitness can be defined as a measure of the reproductive success of a population (Elena and Lenski, 2003), which can be expressed as the natural logarithm of the ratio of the final and initial cell densities of the culture [i.e., No. generations = (log*b* − log*B*)/log2, where *B* = OD of bacteria at the beginning of interval, and *b* = OD of bacteria at the end of time interval; Prescott et al., 1998; Ahn et al., 2006]. Thus, in all other analyses, we used both measures (growth rate and No. generations), allowing the BSocial webtool user to evaluate the importance of one over the other depending on their own application. Biofilm growth was described in arbitrary units of mean number of generations (No. generations) (***n***) and growth rate (***k***).

### Social behavior within mixed populations (BSocial)

In order to assess the function of a strain (in general terms) within a biofilm, relative fitness of either growth rate (*k*) or generations (*n*) were calculated for three data sets for each strain: (_*a*_)—each individual consortia (i.e., 127 consortia; Equations 1 and 4); (_*np*_)—each individual consortia where the strain was not present (i.e., 53 consortia; Equations 2 and 5); and (_*p*_)—all those consortia where the strain was present (i.e., 54; Equation 3 and 6). Thus, relative fitness (*w*) for *n* of each consortium was calculated as:

(1) $$\begin{array}{rcl} w_{a}^{n} & = & \frac{n_{a}}{n_{i}} \end{array}$$

(2) $$\begin{array}{rcl} w_{np}^{n} & = & \frac{n_{np}}{n_{i}} \end{array}$$

(3) $$\begin{array}{rcl} w_{p}^{n} & = & \frac{n_{p}}{n_{i}} \end{array}$$

where *n*_*i*_ is the individual strain's No. generations. The relative fitness for *k* of each consortium was calculated as:

(4) $$\begin{array}{rcl} w_{a}^{k} & = & \frac{k_{a}}{k_{i}} \end{array}$$

(5) $$\begin{array}{rcl} w_{np}^{k} & = & \frac{k_{np}}{k_{i}} \end{array}$$

(6) $$\begin{array}{rcl} w_{p}^{k} & = & \frac{k_{p}}{k_{i}} \end{array}$$

where *k*_i_ is the individual strain's growth rate. Mean results for each consortium were used, and results were drawn using programming language R (R Core Team, 2013) as box plots. In order to observe whether an individual strain had a net effect on the microbial community, we determined whether there was a statistical significant difference (using a *t*-test) between those consortia where the strain was not present ($w_{np}$), and those where the strain was present (). If $\bar{w_{p}}$ > $\bar{w_{np}}$, then we consider the strain to be a “N**et-positive” species**, i.e., a strain which exerted an overall benefit for the community thus increasing *n* or/and *k* when present. Strains that did not present a significant difference between $\bar{w_{p}}$ and $\bar{w_{np}}$, and thus do not bear costs nor present a benefit to the community where denominated **“Neutral” species**. Mean fitness values may be above or below 1. “**Net-negative” species** are organisms that decrease the net productivity ($\bar{w_{p}}$ < $\bar{w_{np}}$) and will most likely have a $\bar{w_{a}}$ > 1. Global net effect definitions are summarized in Table 1. Statistical analysis were performed using program R (R Core Team, 2013; *t*-test, *p* < 0.05).

**Table 1:** **Summary definition of social behaviors**.

**Social behavior**	**Definition**
Positive	$\bar{w_{p}}$ > $\bar{w_{np}}$
Neutral	$\bar{w_{p}}$ = $\bar{w_{np}}$
Negative	$\bar{w_{p}}$ < $\bar{w_{np}}$ or/and all mean values >1

*$\bar{w_{p}}$ —The mean of all consortia data points where the strain is present. $\bar{w_{np}}$ —The mean of all consortia data points where the strain is not present*.

### Phylogeny vs. fitness

The potential relation between consortium phylogenetic composition and fitness was tested. The linear relationship between unifrac distance (Lozupone and Knight, 2005) and fitness were tested by the adonis function within the R vegan package (Oksanen et al., 2013). To this end, first the 16S rDNA gene of the seven strains were aligned by ClustalW (Thompson et al., 1994) and a phylogenetic neighbor-joining tree built by QuickTree v1.1 (Howe et al., 2002). The latter was used to calculate unifrac distance (Lozupone and Knight, 2005) between the different consortium using FastUnifrac (Hamady et al., 2010) and then tested as a function of the fitness by the adonis function (R-Vegan package. Oksanen et al., 2013).

### Effect of increasing species richness on fitness

The fitness data obtained from HT-fitness were processed to describe the effect of increasing diversity on No. generations and growth rate, respectively [BSocial: *Effect of increasing diversity* tab, graphs (a) and (c)]. This analysis allows the user to evaluate whether it was worth using high number of combinations in order to obtain a high growth rate/No. generations. The data sets correspond to 1-species community, or 2-species communities, etc., in a non-cumulative way (Species in community: number of combinations; 1:7, 2:21, 3:35, 4:35, 5:21, 6:7, 7:1). Consortia fitness was normalized by the highest valued individual strain in each category (No. generations or growth rate). Graphs (b) and (d) describe the effect of increasing diversity using the best strains on No. generations and growth rate, respectively. This analysis allows the user to evaluate whether it was worth using just X number of best strains and their combinations to obtain a high growth rate/yield. Individual strain values were ordered from fittest to weakest (1 → 7), and then their combinations were added in order of fittest first in a cumulative manner (Fittest species in community: number of combinations; 1:1, 2:3, 3:7, 4:15, 5:31, 6:63, 7:127). Consortia fitness is normalized by the highest valued individual strain in each category. When fitness is >1, a table showing the consortia reaching this fitness value/s are shown.

### MTBE degradation in the analysis

The individual strains were pre-grown in 5 ml fresh mineral salts medium, centrifuged (14,500 rpm, 1 min), and all strains were adjusted to obtain the same optical density (600 nm) with sterile saline solution (0.9% w/v NaCl). A volume of 1 ml was transferred for each strain to 125-ml glass vials with 25 ml of fresh mineral salts medium amended with 150 mg/L of MTBE, and incubated for 4 days at 30°C under aerobic conditions and controlled agitation (rotation speed, 100 rpm). The glass vials were sealed with Teflon septa. All data are presented as an average of three independent assays, and a control glass vial (without bacteria). The oxygenate concentrations of the gas-tight, teflon-sealed bottles were determined by GC/MS headspace technique as described elsewhere (Purswani et al., 2011). Briefly, a volume of 1.5 mL of the bottle samples were inserted into 2-mL vials and clamped. The samples were heated at 90°C during 90 min, followed by the injection of 50 μL of the gas phase into the GC/MS equipment (Hewlett-Packard 6890 GC coupled to a MS Hewlett-Packard 5973 mass selective detector, Palo Alto, CA, USA). The analysis was performed on a Quadrex capillary column (007-1, dimethylpolysiloxane-PHAT phase, 20 M × 0.18 mm × 6.0 μm). The temperature program was 40°C (3.5 min), 10°C min^−1^ up to 85°C, and 7°C min^−1^ up to 235°C. Helium was used as the carrier gas at a flow rate of 0.4 mL min^−1^ of MTBE was performed using an external standard calibration (*R* > 0.99). All samples were taken in triplicate, and the mean and standard deviations calculated. A significance level of 95% (*p* < 0.05) was selected.

### BSocial webtool

The analyses of these results were mainly performed using the BSocial webtool which is presented here in this paper and freely available to the community. We developed an interactive web application (http://m4m.ugr.es/BSocial.html) using the shiny package in R (RStudio and Inc., 2013). Our web application is designed to be intuitive and have a user-friendly graphical interface (Supplementary Figure 1). Web users can upload a file with their own data and obtain a full analysis of **Net-positive species**, **Neutral species**, and **Net-negative species**, along with other analyses presented in this paper. These analyses are supplemented with downloadable plots, tables, and statistical information. A set of tab panels will guide the user through all the provided results. A complete tutorial can be downloaded from http://m4m.ugr.es/BSocial_tutorial.pdf.

A dataset used in this work is also available at http://m4m.ugr.es/BSocial.html#data and used in the tutorial for illustration purposes. The web application is implemented using R v3.0.2 (R Core Team, 2013) and R packages: shiny v0.8.0, shinysky v0.1.1, R2HTML v2.2.1, and xtable v1.7-1. The web server is Apache v2.2.23 relying on Fedora Linux 64 bit machine.

## Results

### Function and growth

**Figure 1:**
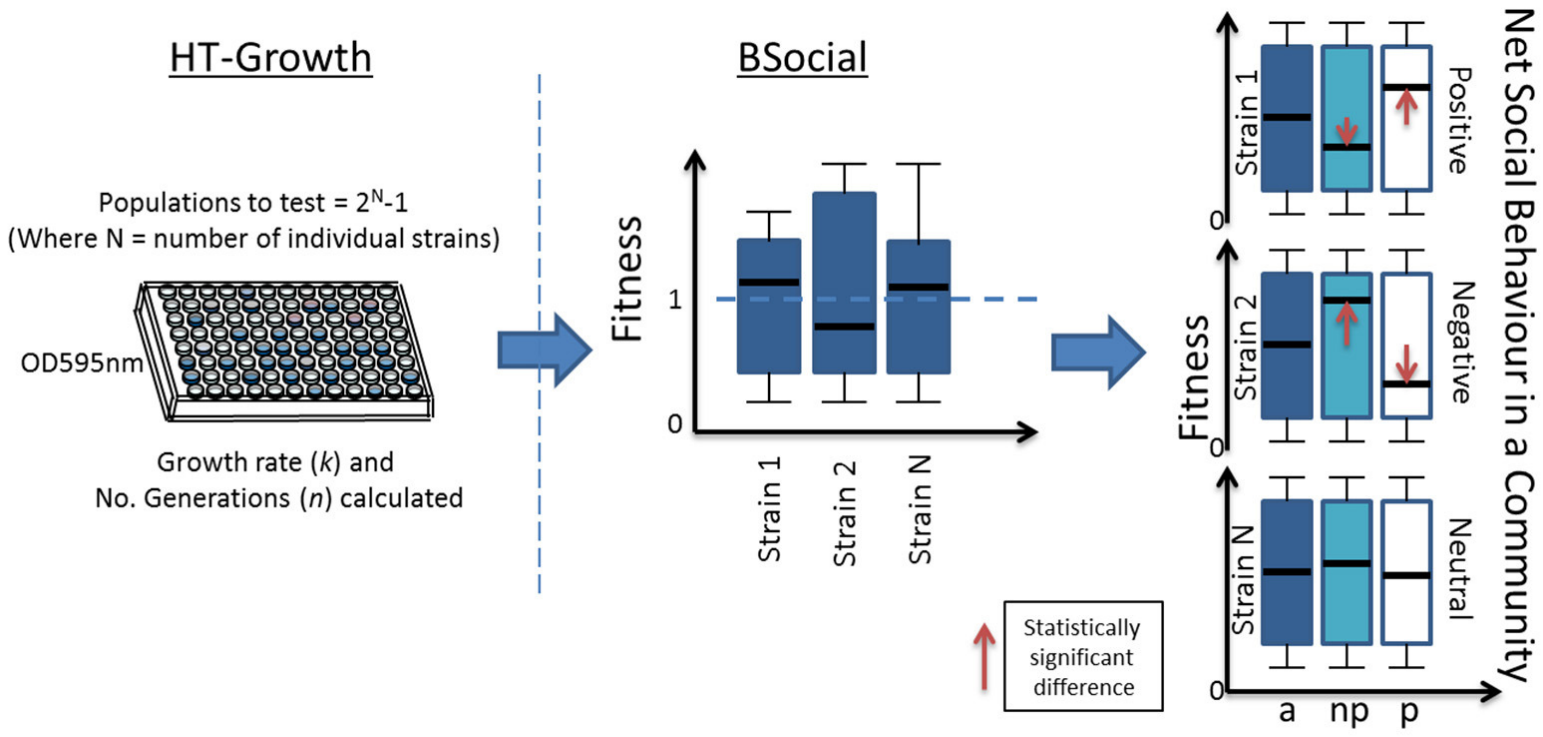
**Deciphering social behavior using the combination of HT-Growth and BSocial methods**. The number of populations depend on the number of individual strains, and are grown on microtiter plates with continuous OD readings at regular intervals, enabling *n* (number of generations) and *k* (growth rate) calculations. All the data points (*a*) are normalized with each individuals *n* or *k*. Data points are then separated according to absence (*np*) or presence (*p*) of the individual strain in the population. Statistical differences between the means of *np* and *p* will return a net positive or negative social behavior, whereas a non-difference will determine the strain to have a neutral social behavior.

The HT-Growth and BSocial analyses were performed on species with varying growth and degradation abilities on fuel ethers MTBE and ETBE. A summary of both methods is observed in Figure 1. A total number of 127 combinatorial populations were grown during a 4-day period on 96-well plates from seven different strains, with OD measurements at 1 h intervals. The No. generations and growth rate were determined for each population, and this data was run in BSocial for further analyses. The mean results of triplicate assays were plotted (No. generations vs. Log phase time) and can be observed in Supplementary Figure 2.

On the BSocial website, the top ten consortia on the *Growth* tab, are chosen firstly by highest No. generations, and then lowest log phase time. The top three consortia suggested for our two conditions were: MTBE—strain SH7, strain MS2, and consortium MS2/SH7; and for ETBE consortia—EE6/MS2, MS2/SH7, and strain SH7. MTBE degradation was tested for all 127 cultures after a 4-day incubation period. Generally, positive correlation between growth (*n* and *k*) and oxygenate degradation were observed (Supplementary Figure 3), however the highest *n-* or *k*-valued populations did not correspond with the most efficient MTBE degrader culture. Unique use of growth observation on a xenobiotic compound will not render the best degrader consortium.

### Social behaviours within communities (BSocial)

**Figure 2:**
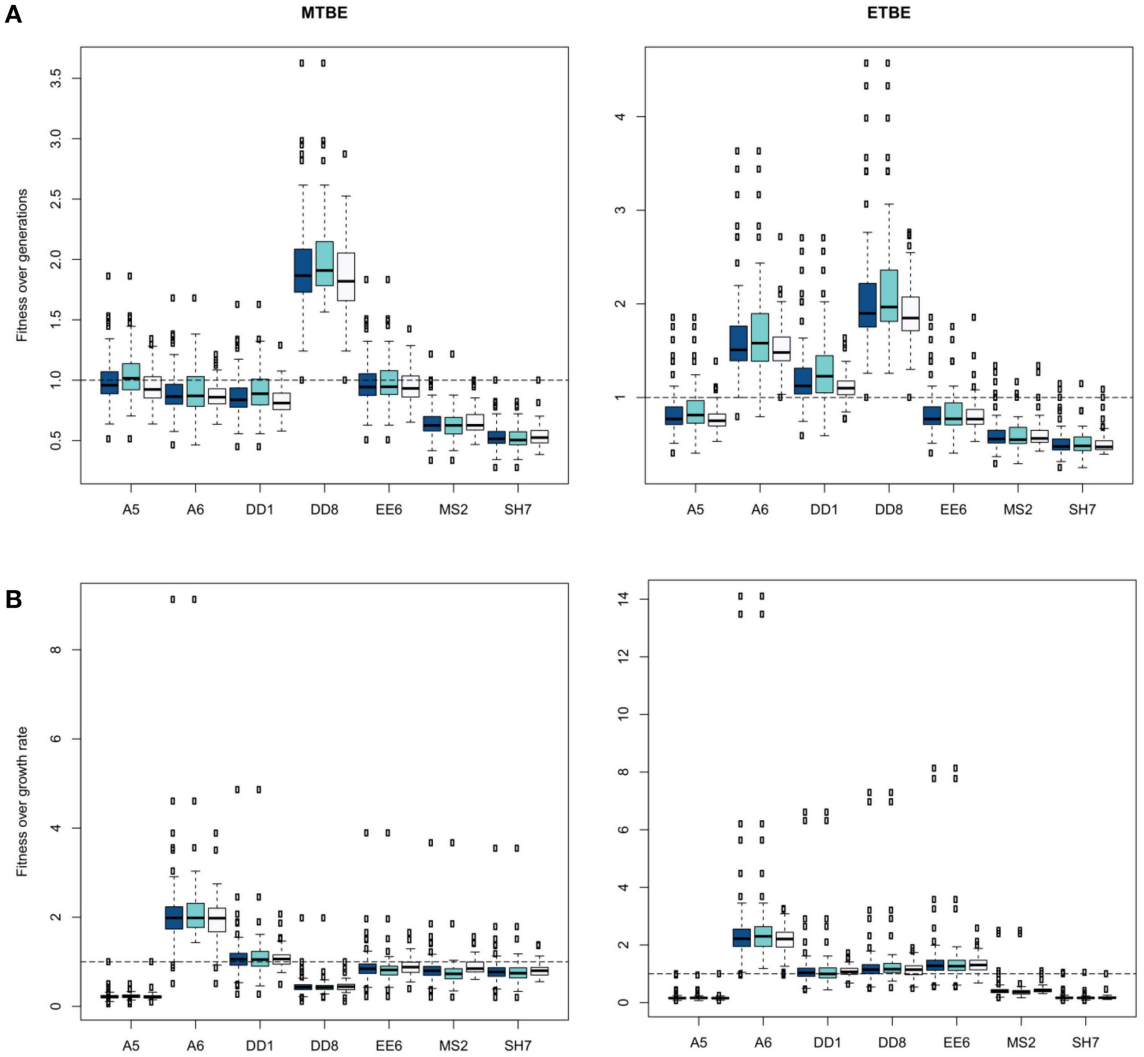
**Box plot representation of fitness data for the assignation of net-positive species, no net effect species, and net-negative species via $\bar{w_{p}^{n/k}}$ and $\bar{w_{np}^{n/k}}$ comparative using (A)** No. generations and **(B)** growth rate data for each strain. Three box plots assigned to each strain contain the fitness values: 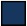
—all consortia data points; 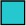
—all consortia data points where strain is not present; and 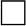
—all consortia data points where strain is present. The strain whose median box plot values fall >1 fitness, are assigned the social behavior “Net-Negative.” The box's height spans between the interquartile range (IQR, 25th and 75th percentiles), and whiskers extend to 1.5-fold the IQR. Outliers beyond the whiskers are plotted as open circles.

Within the BSocial analyses, normalization of each culture was performed with each individual strain, thus the fitness observed for each data set (*a, np, p*) is a general view of the individuals possible contribution to a mixed community. Summarized in Table 2 are the social behavior assignations of each strain for each condition, using No. of generations or growth rate in MTBE and ETBE (applying the BSocial: *Social behavior* tab). The $\bar{w_{a}}$ -values for strains DD8 (Figure 2A) and A6 (Figure 2B) were always >1 in all three conditions, thus these strains are **net-negative species** since they always benefit in the presence of others. Furthermore, when present in consortia, they exert a negative effect on the consortia, since $\bar{w_{p}}$ < $\bar{w_{np}}$. Even though some strain mean values were not >1, they exerted a negative effect on mean values when present, thus these too were classified as **net-negative species**. On the other hand, MS2 exerted a **net-positive effect** over strains in the growth rate mean values for MTBE, and EE6 and SH7 were **neutral effect species** when both data sets (i.e., No. generations and growth rate) were observed. Since the biochemical pathway of MTBE and ETBE degradation are the same (Chauvaux et al., 2001; Lopes Ferreira et al., 2006; Schuster et al., 2013) the results on both were joined. Thus overall, we would not suggest any user to use net-negative strains in their consortia if they appeared on either data set, and would suggest using EE6, SH7, and definitely MS2 for bioremediating biofilm-based technologies. If we choose a consortia containing **net negative species** and **no net effect species** e.g., A5-A6-DD1 in MTBE, and compare these under the same conditions with the recommended strains in consortia, i.e., EE6-MS2-SH7, the first consortia should perform worse than the latter consortia. We can corroborate the latter, since consortia A5-A6-DD1 has a mean growth rate of 0.102 generations/h and a mean No. generations of 3.2, whereas consortia EE6-MS2-SH7 has a mean growth rate of 0.18 generations/h and a mean No. generations of 4.1. Consortium EE6-MS2-SH7 contributed to the highest recorded degradation (81% MTBE removal) whereas A5-A6-DD1 contributed to a low degradation (20% MTBE removal). The median degradation percentage of communities that only contained positive and/or neutral strains (EE6, MS2, SH7) strains was significantly (*p* < 0.002) higher than the median of communities that only contained net-negative strains (28 and 16% MTBE removal, respectively). Moreover, a consortium of our three candidates plus the negative contributor DD1 rendered the lowest MTBE removal (0.14%).

**Table 2:** **Assignation of social behaviors according to *t*-test (*p* < 0.05) of fitness values shown in Figure 2, using mean $w_{p}^{n}$ - and $w_{np}^{n}$ -values, or $w_{p}^{k}$ - and $w_{np}^{k}$-values**.

		**Social behavior**	
	**Strain**	**MTBE**	**ETBE**
Number of generations	A5	Negative	Negative
	A6	Neutral	Negative
	DD1	Negative	Negative
	DD8	Negative	Negative
	EE6	Neutral	Neutral
	MS2	Neutral	Neutral
	SH7	Neutral	Neutral
Growth rate	A5	Neutral	Neutral
	A6	Neutral	Negative
	DD1	Neutral	Neutral
	DD8	Neutral	Negative
	EE6	Neutral	Neutral
	MS2	Positive	Neutral
	SH7	Neutral	Neutral

### Phylogeny vs. fitness

Hamilton's “Genetic relatedness” describes the possibility of altruistic actions between genetically related individuals. So perfect cooperation has been described as a biofilm of a single strain, thus having high genetic relatedness (Foster et al., 2007), since cells with the same genetic background will not have evolutionary conflicts of interest. In order to test whether we could observe this relation in our experiments, maximum phylogeny distance between the consortiums' strains was tested against fitness with the adonis function, resulting in no correlation between the phylogenetic composition of the consortia and their fitness (*R*_2_ < 0.04; data not shown). One clear example is the non-increase in fitness or degradation in the 2-strain consortium of *R. ruber* A5 and *R. ruber* EE6 compared to their individual fitness.

### Effect of increasing diversity

**Figure 3:**
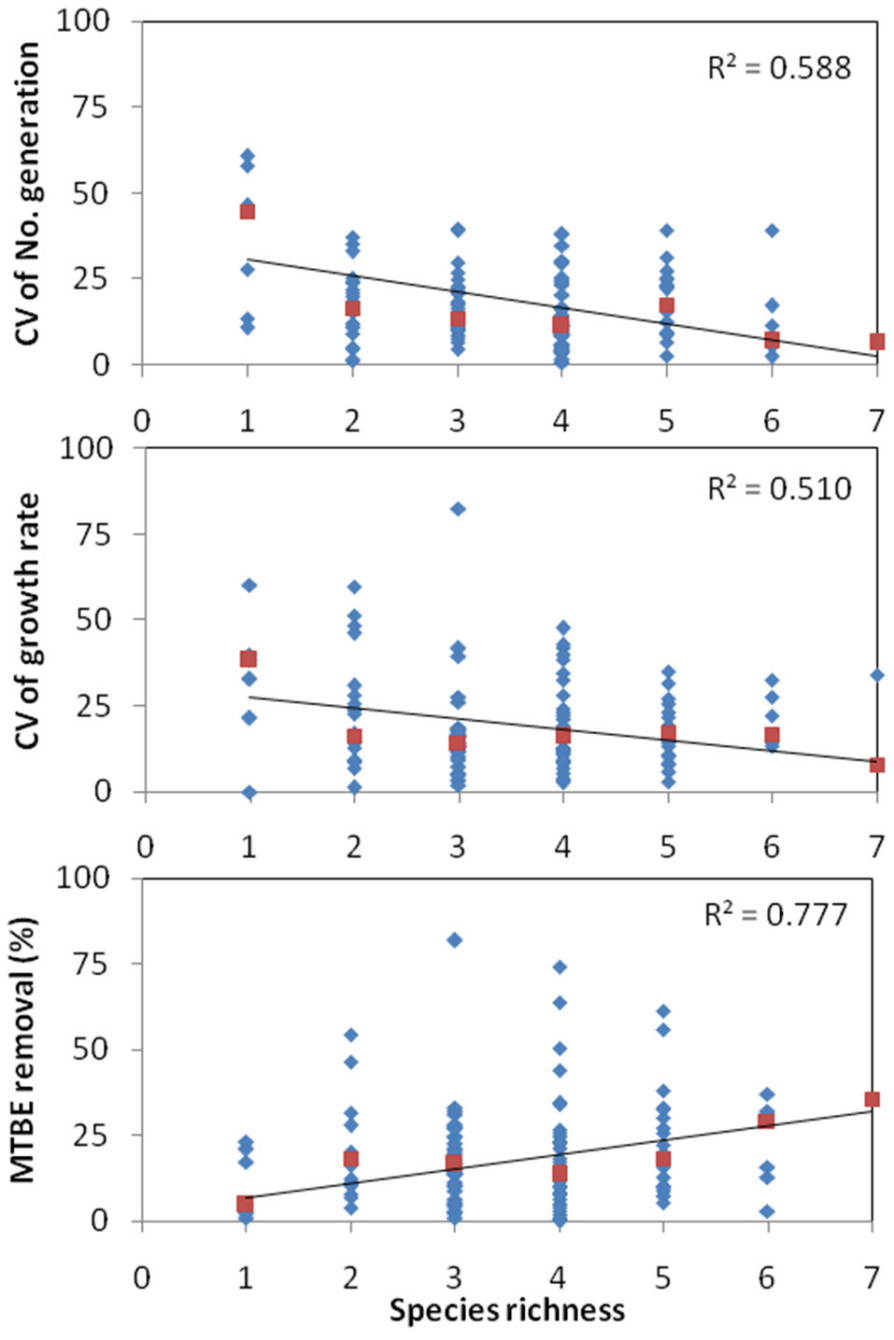
**Effect of coefficient of variation (CV) of total community fitness (number of generations or growth rate) or function (MTBE removal) on microbial species diversity**. The data shown is for MTBE grown communities. The lower coefficients of variation in rich diverse systems describe the stability of fitness or function by diversity. 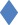
—CV of community, 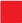
—Median CV for *N* species.

The coefficient of variation (CV) was calculated as an indicator for community stability with increasing species richness as in Tilman et al. (1996). Increasing stability was described with decreasing variance against increasing species diversity. Using this same principal, we determined the effect of increasing species diversity upon fitness variance and function (Figure 3). We can observe minor negative correlations in both CV for *n* (*r*^2^ = 0.58) and CV for *k* (*r*^2^ = 0.51) with increasing species richness. Moreover, positive correlation in function (*r*^2^ = 0.77) was observed with increasing species richness.

**Figure 4:**
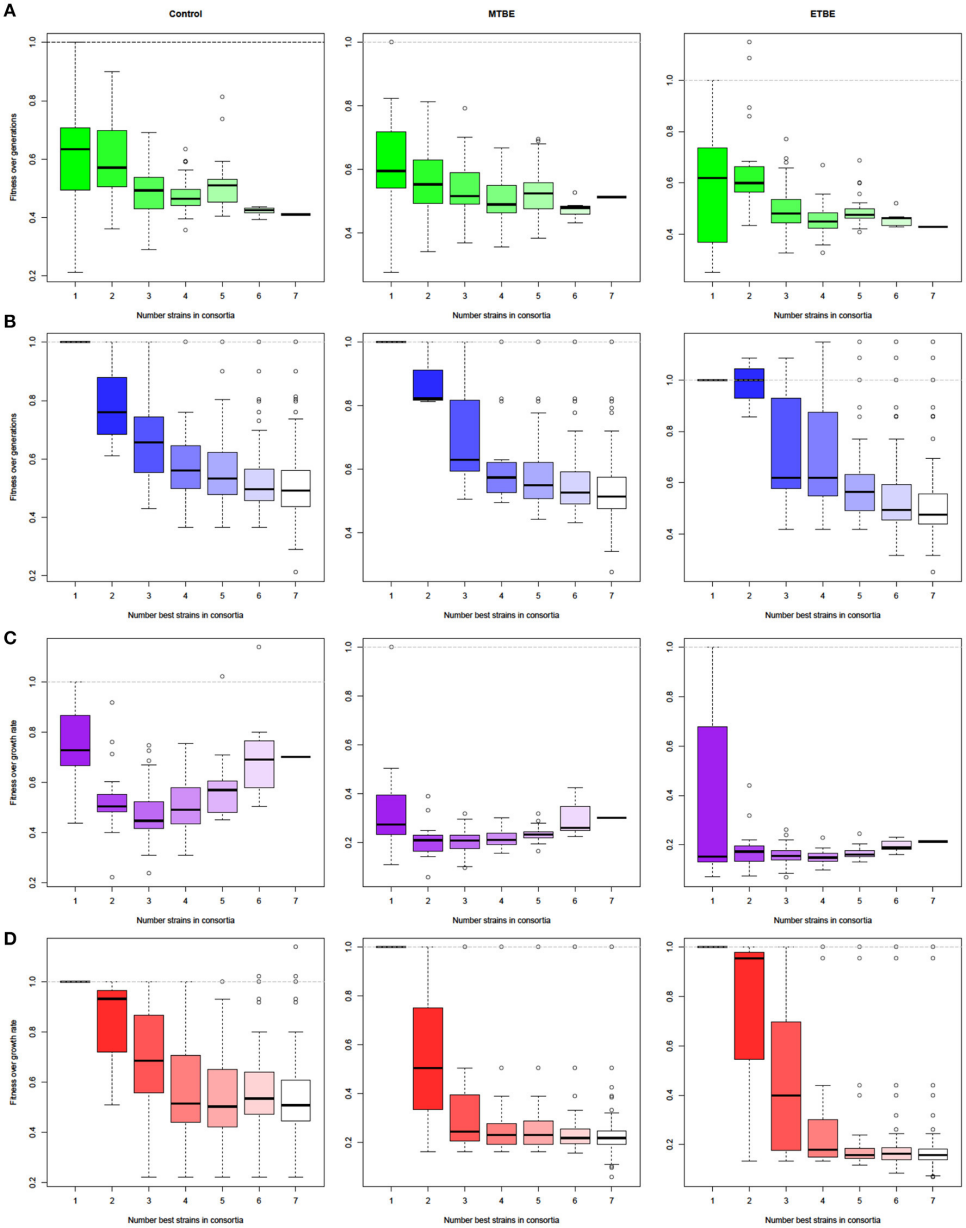
**Effect of increasing species richness on fitness**. General decrease in fitness is observed with increasing species richness **(A,C)**. Data points were normalized by the fittest species (non-cumulative analysis). Panels **(B)** and **(D)** show how many fittest species (No. of best strains) are needed to obtain the fittest combination, where any data point >1 is better than the fittest individual strain. Data points were normalized by the fittest species, adding each species (previously ordered from fittest to weakest, 1 → 7) and all combinations cumulatively. No. generations **(A,B)** or growth rate **(C,D)** data were used. The box's height spans between the interquartile range (IQR, 25th and 75th percentiles), and whiskers extend to 1.5-fold the IQR. Outliers beyond the whiskers are plotted as open circles.

Optimal selection of cultures with increasing diversity was tested (BSocial: *Effect of increasing diversity* tab) in order to evaluate: (a) fitness with increasing species richness (Figures 4A,C), and (b) the number of fittest individual strains (ordered from fittest to weakest, 1 → 7) needed to obtain the fittest inoculum, in a cumulative analysis (Figures 4B,D). In both cases, the data points were normalized by the fittest individual strain. Thus, any data point higher than 1, accounts for reasonable testing in those numbers. As can be observed in graphs, general decrease trend in fitness is observed with increasing species richness (Figures 4A,C). Furthermore, pair combination of the seven strains (Figure 4A:ETBE) or all combinatorial cultures for up to four of the best individual strains(Figure 4B:ETBE) would have accounted for a highest biomass production culture. However, if we would have used only two of the best strains in ETBE and tested their combinations, we would have fallen short of achieving the combination with the highest fitness values (Figure 4B).

## Discussion

### Application of BSocial on fuel ether bioremediation

The main analytical tool in BSocial is the ability to decode the net impact of an individual species over a large number of mixed populations, thus ultimately deciphering its social behavior in that specific environment. Furthermore, its use in bioremediation has been demonstrated.

The microbial species used in the study were isolated from different backgrounds (contaminated soil, water, and a bioremediating biofilter), with different abilities for MTBE bioremediation. Consequently, not having evolved and established stable relationships previously, this work describes their first social interactions. Deciphering the net social behavior of individual strains resulted in the choosing of one positive and two neutral strains (Table 2), discarding those that were labeled net negative strains in either carbon source (MTBE or ETBE). Consequently, the consortium turned out to be the most efficient degrading consortium (EE6-MS2-SH7), A significant increase in median function occurred only when communities with positive and neutral socially assigned strains were used, i.e., a 1.75-fold median increase in MTBE removal compared to communities with socially negative assigned strains. Therefore, there is a clear relationship between the use of positive/neutral strains and higher MTBE degradation. In previous work (Catón-Alcubierre, 2013; Guisado et al., 2013), we found that the strains EE6, MS2, and SH7 were apt for a biofilm-based bioreactor, but this was tedious and time-consuming since many factors were to be weighed in (MTBE removal, growth, growth on support material, production of EPS, and/or adhesin appendages), and were only determined for 2-species communities. Though we do not discard the use of such tests, our high throughput method and BSocial analysis can redirect the user toward a quick and efficient testing for inoculum selection. Could HT-growth data (number of generations or growth rate) exclusively deliver the best degrader populations?

Sole choice of the best HT-Growth strain (Supplementary Figure 2) would not have selected the highest degrading consortium. The main reason why this occurs is the use of available nutrients other than MTBE, which may include perished bacteria or the use of degrader exudates. Thus, the combined work between HT-Growth method and BSocial analyses renders this a useful tool to apply on understanding highly diverse growth-dependant systems.

### Effect of diversity on community stability

Community stability, defined as defying change or rebound from change, is a requisite for optimum microbial community function. The use of more than one strain within a biofilm for a bioremediating process is recommended, since contamination in bioreactor systems will most likely occur, possibly resulting in a markedly decrease in the functional species. The stability-diversity hypothesis predicts more stable communities as diversity increases (McGrady-Steed et al., 1997), but more complex relations could exist between diversity and stability (Pfisterer and Schmid, 2002). Our results do infer a general tendency compliant with the stability-diversity hypothesis since we observe decreasing fitness variance (CV*n* and CV*k*) with increasing species richness (Figure 3). This trend has also been observed in other microbial studies, testing predator production (Saleem et al., 2013) and nutrient consumption (Saleem et al., 2016) with increasing bacterial richness. The function, as MTBE removal, was also positively correlated with an increase in species diversity. In all three cases, **the overall effect of increasing diversity leads to a more stable MTBE degrading community**. For more information on community stability, further work testing resistance and resilience to disturbance could be performed.

### Effect of diversity on fitness

The effect of species richness on fitness was evaluated for No. generations and growth rate (Figures 4A,C, respectively). The decrease in fitness with increasing species richness observed in Figure 4A is consistent with results shown in Becker et al. (2012). In the latter study, the antagonistic interactions increased with species richness, thus rendering their community functions (root colonization and plant protection) close to obsolete. Thus, a balance between increasing community stability and decreasing function should be taken into account when increasing species richness. The number of strains and combinations necessary to obtain highest fitness values were observed in Figures 4B,D, in order to evaluate whether the species richness falls short in the study or not. In all cases, combinations of up to a species richness of four was enough to obtain the fittest consortia.

Overall, interactions that englobe cooperation, commensalism, and exploitation, whether the focal species (actor) in question is benefiting both itself and the community (recipient), or benefits the community through metabolite secretion, or benefits the community whilst being harmed (or viceversa), results in a species increasing productivity in the form of growth (Net-positive strain). The latter is true as long as the positive effects outweigh the negative effects from a net-negative strain. Neutral effect does not exactly mean that there is no effect on the whole community. In the latter situation, adding the focal species implies some fitness loss on the community as the focal species will be taking resources from them in order to grow, however, it could also outweigh in equal strength the negative effects of other species. Thus overall, we describe net positive and neutral effects to be held by synergistic interactions, and net negative held by antagonistic interactions. Synergistic interactions may be based on cooperation interactions, but cooperation is rare among completely unrelated species (Foster and Bell, 2012; Rakoff-Nahoum et al., 2016) especially if they have not evolved together, such as our populations. Thus, our positive and neutral strains (EE6, MS2, and SH7) have synergistic effects over MTBE removal.

A study on how biodiversity affects stability of cooperation (Jousset et al., 2013) used highly related microorganisms (eight *Pseudomonas* spp. genotypes) to determine whether genetic relatedness promotes the ability to cooperate (positive/neutral strains) predicted via community phylogeny history, or if non-additive effects drive defectors (negative strains), community richness is expected to predict stability of cooperation. They observed that evolutionary history predicted the stability of cooperation between intra-multispecies communities, though they take into account that closely related species may share the same niche and same nutrients which may increase competition. However, our data suggests that only non-additive effects come into play in community stability, since there is no effect between phylogeny and fitness.

### Social evolution

Evolution of cooperation is strongly influenced by population structure (Celiker and Gore, 2013) and according to kin selection theory, that infers cooperative interactions between closely related individuals (Hamilton, 1964). The r in Hamilton's rule for a cooperative trait to spread or not is the relatedness of the gene of interest, and this in turn may correlate with global relatedness at the genome level. We observed the latter not to be true in our bioremediation system. Minimal phylogenetically distant consortia did not correlate with an increase in fitness (*R*^2^ < 0.04). Xenobiotic degrading pathways tend to exist in mobile elements, thus ensuring greater response from a community in the event of emerging chemicals (Kolvenbach et al., 2014). The observation of phylogenetic distance on function may differ if we were to observe the effect of biological nitrogen fixation, where phyla and function are highly related.

Biofilms in social evolution theory (Nadell et al., 2009), predict that slow-growing high-yield strain will outcompete a fast-growing low-yield strain since biofilms composed solely of slow-growing high-yield cells produce more biomass than biofilms composed of fast-growing low-yield cells. In a mixture, fast-growing cells will outcompete slow-growing cells within a biofilm through direct competition. According to Nadell et al. (2009), “bacteria slow their growth to use resources efficiently and secrete molecules that benefit other cells in their surroundings.” Therefore, even though EE6, MS2, and SH7 may not have the highest growth rates, this may actually be advantageous in a cooperating biofilm structure with desired long-term social interactions.

## Conclusion

The approaches used in this study (HT-Growth and BSocial analyses) can be extended to test and develop ideal combinations of degrader strains for applied microbial biotechnological purposes, such as bioremediation. Biofilms are the causative agents for many persistent bacterial infections (Costerton et al., 1999) as well as industrial problems associated with biofouling (Jass and Walker, 2000), thus finding the key cooperating bacterial strain/s within a biofilm allows for active-targeting of the most relevant species (e.g., **net-positive species)**, thus overall weakening the biofilm. The **net-positive species** strain/s may well-contribute to quorum sensing, or be highly active in the transference of resistance genes to others, or be the main compositor that holds the biofilm together through its EPS production thus protecting and anchoring other microorganisms. Thus, understanding the species social behavior in biofilms can reveal the dynamics of a microbial community, where action can be taken toward specific biofilm members, either to enhance their social behavior (such as incrementing commensal gut bacteria) or to reduce and eliminate its participation (such as tooth decaying bacteria). BSocial is thus an excellent platform for optimizing biotechnological processes (like the one shown in this paper), and testing ecological and evolutionary questions.

## Authors contributions

JP, JG, and CP were responsible for the conception of the study. JP and IG performed all measurement experiments. JP, RR, and AM are responsible for designing and interpreting the analytical tools in BSocial. RR designed the webtool. JP, RR, AM, and CP drafted the manuscript and JG and IG contributed to the final manuscript.

### Conflict of interest statement

The authors declare that the research was conducted in the absence of any commercial or financial relationships that could be construed as a potential conflict of interest.

## Abbreviations

MTBE: methyl *tert-*butyl ether
ETBE: ethyl *tert*-butyl ether
EPS: extracellular polymeric substance
*n*: number of generations
*k*: growth rate.
