# Supplementary material for: BSocial: Deciphering Social Behaviors within Mixed Microbial Populations
Source: Front Microbiol. 2017 May 24;8:919. doi: 10.3389/fmicb.2017.00919 (PMC5442188; doi:10.3389/fmicb.2017.00919)
Supplement: Supplementary file 1 [file Image1.PDF]

Supplementary Material

BSocial: Deciphering Social Behaviours within Mixed Microbial Populations

Jessica Purswani*, Rocio C. Romero-Zaliz, Antonio M. Martín-Platero,

Isabel M. Guisado, Jesús González-López, Clementina Pozo.

*** Correspondence:** Corresponding Author: jessicapurswani@ugr.es

1 Supplementary Figures and Tables

1.1 Supplementary Figures

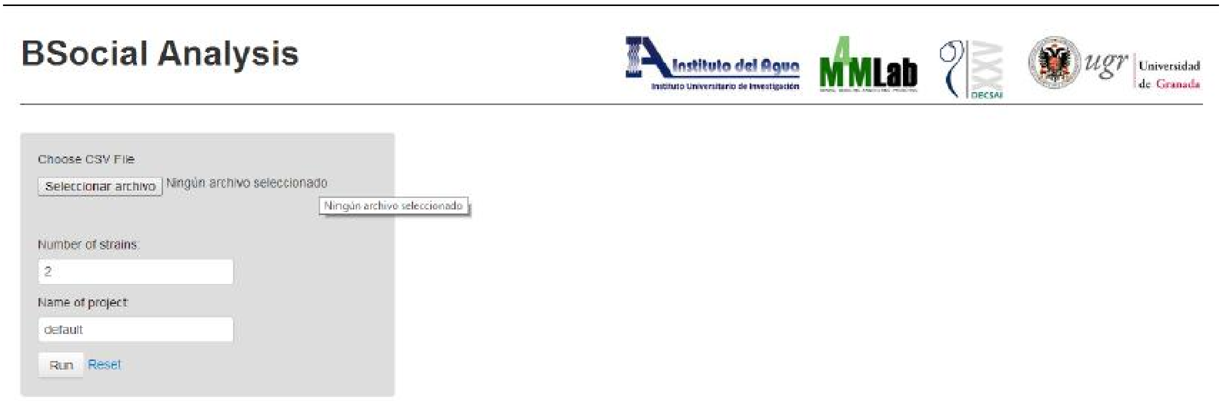

Supplementary Figure 1. Screenshot of the BSocial web server application

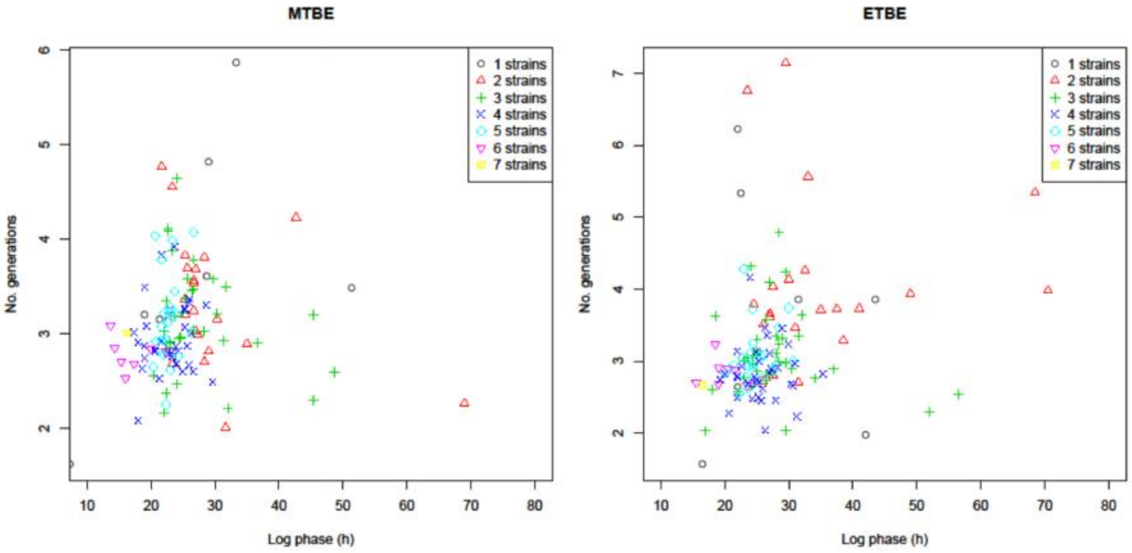

Supplementary Figure 2. Growth (as No. generations) of the microbial consortia during a 4-day period colour coded by number of strains on different carbon sources a) MTBE b) ETBE. The best consortia are those with the highest No. generations followed by the lowest Log phase time.

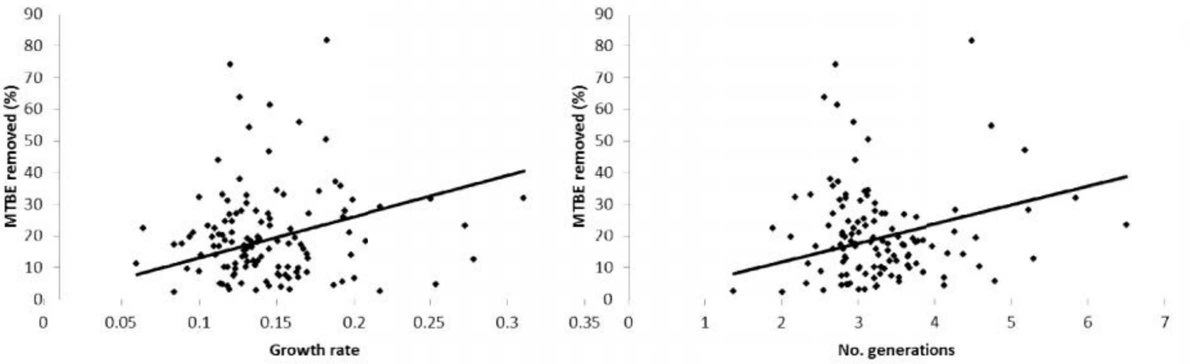

Supplementary Figure 3. Positive tendency of growth rate and no. generations with percentage of MTBE degraded.
